# Effectiveness and therapeutic validity of physiotherapeutic exercise starting within one year following total and unicompartmental knee arthroplasty for osteoarthritis: a systematic review

**DOI:** 10.1186/s11556-023-00317-4

**Published:** 2023-03-29

**Authors:** Amarins Koster, Martin Stevens, Helco van Keeken, Sanne Westerveld, Gesine H. Seeber

**Affiliations:** 1 Department of Orthopaedics, University of Groningen, University Medical Center Groningen, Groningen, The Netherlands; 2 Department of Human Movement Sciences, University of Groningen, University Medical Center Groningen, Groningen, The Netherlands; 3 University Hospital for Orthopaedics and Trauma Surgery Pius-Hospital, Medical Campus University of Oldenburg, Oldenburg, Germany

**Keywords:** Knee prostheses, Rehabilitation, Joint replacement, Physical therapy, CONTENT scale

## Abstract

**Background:**

To determine the effectiveness and therapeutic validity of physiotherapeutic exercise after total and unicompartmental knee arthroplasty for osteoarthritis. It was hypothesized that interventions of high therapeutic validity result in superior functional recovery after total and unicompartmental knee arthroplasty versus interventions of low therapeutic validity.

**Methods:**

A systematic review incorporating a comprehensive database search of five major databases relevant to the topic was conducted. Randomized controlled trials were reviewed if they included studies that compared postoperative physiotherapeutic exercise with usual care or compared two types of postoperative physiotherapeutic interventions. All included studies were assessed for risk of bias (using the Cochrane Collaboration’s tool) and therapeutic validity (using the Consensus on Therapeutic Exercise Training scale). The characteristics of the included articles and their results on joint and muscle function, functional performance, and participation were extracted.

**Results:**

Of the 4343 unique records retrieved, 37 articles were included. Six of them showed good therapeutic validity, suggesting low therapeutic validity in 31 studies. Three articles showed a low risk of bias, 15 studies scored some concerns for risk of bias and 19 studies scored high risk of bias. Only one article scored well on both methodological quality and therapeutic validity.

**Conclusion:**

Due to heterogeneity of outcome measures and length of follow-up, as well as limited reporting of details of the physiotherapeutic exercises and control interventions, no clear evidence was found on effectiveness of physiotherapeutic exercises after total and unicompartmental knee arthroplasty. Homogeneity in intervention characteristics and outcome measures would enhance comparability of clinical outcomes between trials. Future studies should incorporate similar methodological approaches and outcome measures. Researchers are encouraged to use the Consensus on Therapeutic Exercise Training scale as a template to prevent insufficient reporting.

**Supplementary Information:**

The online version contains supplementary material available at 10.1186/s11556-023-00317-4.

## Background

Osteoarthritis (OA) is the most common joint disorder globally, whereby OA in the knee alone accounts for almost four-fifths of the worldwide OA burden [1]. Primary OA-related symptoms include joint pain and stiffness, which can substantially impact patients’ functioning and quality of life (QOL) [1, 2]. The primary risk factor for developing OA is older age. As life expectancy rises, so does the incidence of OA [3]. Knee OA management should first and foremost follow a non-surgical approach. A surgical procedure is indicated only when pharmacological and non-pharmacological conservative treatment options fail. Based on the damage to the knee, a unicompartmental knee arthroplasty (UKA) or a total knee arthroplasty (TKA) is chosen [4–6]. Numbers for both TKA and UKA have increased significantly in recent years. While 19,521 TKAs and 1,586 UKAs were performed in the Netherlands in 2011, as many as 21,444 TKAs and 5,648 UKAs were performed in 2021 [7]. A similar trend can be observed in other Western countries: for example, in Germany 103,882 TKAs and 15,011 UKAs were registered in 2018, and 107,596 TKAs and 16,831 UKAs in 2019 [8, 9].

Physiotherapeutic exercises play a significant role in optimal TKA/UKA rehabilitation. Exercises may include strengthening, endurance, flexibility, and balance exercises that aim to correct impairments, restore muscular strength, and joint range of motion (ROM), and ultimately improve subjects’ physical health and restore normal function [10]. According to the International Classification of Functioning, Disability and Health (ICF) model, the functioning of an individual can be described at three levels: body functions and structures, activities, and participation [11]. It is known that patients can still have deficits in body functions and structures (e.g., quadriceps muscle strength and postural stability deficits, weight-bearing asymmetry) and activities (e.g., reduced walking and stair-ascent speed) up to two years after TKA [12–15]. The effects of physiotherapeutic exercises depend, among other things, on exercise intensity, duration, frequency, and length of time after surgery [12]. Determining the effects of physiotherapeutic exercises is not straightforward, as exercise can be delivered and performed in several ways and settings, possibly creating heterogeneity.

Previous systematic reviews have been devoted to the effectiveness of physiotherapeutic exercise after TKA/UKA, but conclusions drawn from these are ambiguous. Lowe et al. (2007) found only a small-to-moderate effect of the short-term benefits of functional exercises [16], yet the systematic review and meta-analysis of Pozzi et al. (2013) concluded that physiotherapeutic exercise is effective when combining strengthening and intensive functional training using land-based or aquatic programmes [17]. However, these studies lack a sufficiently detailed description of the physiotherapeutic interventions applied in the included primary studies. For example, interventions can be delivered in quite different ways across trials (e.g., regarding exercise timing, intensity, progression monitoring and subsequent adjustments, exercise personalization and contextualization) [18]. It is reasonable to expect that the way in which exercises are delivered as well as specifications of the individuals to whom those are delivered can influence their effectiveness. In addition, responses to exercise are determined by the mode, frequency, intensity, and duration of exercise. In practice, the dose is also determined by adherence to the programme. The effects of exercise programmes observed in clinical trials are likely to vary because trials use different training doses and inspire different levels of adherence [19]. Sometimes interventions are even not described at all, for example in the systematic review of Fatoye et al. [20]. It has been shown that the quality of the intervention used can influence rehabilitation outcomes [18, 19]. However, without any determination of the content and therapeutic validity of the physiotherapeutic interventions used in the different articles informing the systematic reviews above, it becomes impossible to conclude from these papers which type of physiotherapeutic exercise intervention would serve TKA/UKA rehabilitation best.

To draw valid conclusions about the efficacy of physiotherapeutic exercises, it is recommended to examine an intervention’s effectiveness and its therapeutic validity in systematic reviews. Therapeutic validity is defined as the potential effectiveness of a specific intervention given to a potential target group of patients [19]. To determine degree of therapeutic validity, Hogeboom et al. developed the Consensus on Therapeutic Exercise Training (CONTENT) scale [20]. This scale has been used in systematic reviews examining exercise interventions for fibromyalgia, chronic obstructive pulmonary disease, heart disease, and joint replacement populations [20–24]. It has not yet been applied in TKA/UKA rehabilitation.

This study therefore aims to determine the effectiveness and therapeutic validity of physiotherapeutic exercise following TKA/UKA for OA. It is hypothesized that interventions of high therapeutic validity result in superior functional recovery after TKA/UKA versus interventions of low therapeutic validity.

## Methods

A systematic review including a narrative synthesis. The protocol was registered with PROSPERO (CRD 42022311661) before study initiation. Study preparation, conduct, and reporting followed the Preferred Reporting Items for Systematic Reviews and Meta-Analyses (PRISMA) statement (S1) [25].

## Eligibility criteria

Study inclusion and exclusion criteria were predefined using the PICOS (Patient, Intervention, Comparisons, Outcome, Study Design) approach [26]. This resulted in the following criteria:

- Patient: we included studies investigating patients who underwent unilateral primary UKA or TKA due to OA. Informing about bilateral TKA/UKA or kneecap replacement precluded a study’s inclusion.
- Intervention: studies investigating active land-based or water-based physiotherapeutic exercises (in inpatient and/or outpatient settings) starting within 12 months following TKA/UKA were included. Studies investigating passive physiotherapeutic modalities such as manual therapy, massage, osteopathy, and electrical stimulation, as well as studies investigating preoperative physiotherapy, were regarded as ineligible.
- Comparison: studies comparing postoperative physiotherapeutic exercise versus usual care or two types of active postoperative physiotherapeutic interventions were deemed eligible.
- Outcome: studies were included if at least one of the following outcome categories was reported: joint and muscle function (corresponding with the ICF model’s “body functions”, e.g., strength, range of motion [ROM]), functional performance (corresponding with the ICF’s model “activities”, e.g., walking speed), and subjective patient self-reported outcomes (corresponding with the ICF’s model “participation”, e.g., questionnaires evaluating general and disease-specific quality of life; pain scales). Reported outcome measures’ effects on the acute postoperative phase (e.g., hospital length of stay, wound leakage) and/or measures of specific muscle properties (e.g., morphology, architecture) were not deemed eligible.
- Study design: to be included, studies had to follow a randomized controlled design, and pre- and post-intervention measurements had to be conducted for the intervention and control groups so that results were available for both groups. Studies not following a randomized controlled design, abstracts/conference proceedings, editorials, and letters to the editor were excluded.

## Search Strategy

Search terms were developed using the PICOS mnemonic [26]. Next, database-specific search strings were established with the help of an experienced scientific librarian at University Medical Center Groningen. Last, a combination of medical subject headings (MeSH) and/or concepts subject headings based on the target database and keywords/text words were meaningfully linked. The search strings used in each database are presented in S2. The search was limited to articles published after 1999 and in English only. The rationale for these limitations was (1) to preclude outdated surgical techniques for TKA/UKA and subsequent rehabilitation protocols and (2) English was the only language all involved reviewers were proficient with. All databases were searched for eligible articles on 14 February 2022.

## Information sources

Relevant scientific literature was identified in online databases pertinent to the topic: AMED (via EBSCO host), CINAHL (via EBSCO host), Cochrane Trials (via Cochrane Library), Embase (via embase.com), and MEDLINE (via OVIDSP). All databases were accessed via University Medical Center Groningen.

## Study selection

All retrieved studies from the different databases were exported into the review manager software Covidence (Veritas Health Innovation Ltd, Melbourne, Australia), which automatically excluded all duplicates. One reviewer (GHS) cross-checked that automatic deduplication for correctness. A pilot screening was performed to ensure that both reviewers’ agreement during study selection met at least 75% as per previous recommendations [27]. To this end, two reviewers (AK and SW) independently screened the first 50 studies’ titles and abstracts. Both reviewers’ pilot screening agreement reached 96%. Only two conflicts occurred, which were solved during a consensus meeting between the same two reviewers. Next, a three-reviewer model was employed wherein the two researchers (AK and SW) independently screened all remaining articles by title and abstract for eligibility. In case of unsureness as to whether to include or exclude an article just by reading the title and abstract, the study was included for full-text review. Studies without an accompanying abstract were included if the title did not clearly suggest ineligibility. Next, the full texts of all studies deemed eligible during the title and abstract screening phase were retrieved and uploaded into Covidence. The same two researchers subsequently read the full texts of the remaining articles to ensure their eligibility. Disagreements between the individual judgments were discussed after each respective step to achieve consensus. In case of no consensus, the conflict was resolved by a third blinded reviewer (GHS or MS).

## Data extraction and quality assessment

Two reviewers (AK and SW) independently extracted the data from all included full-text articles using the Covidence software. Before establishing the final data extraction template in Covidence, a custom-built data extraction sheet was created using Microsoft Excel (Version 2204; Microsoft Cooperation, Washington, USA) and piloted by the two reviewers using two randomly picked articles on the same topic but including total hip arthroplasty patients to validate the template’s operational utility and to refine as necessary. Once finalized, the data extraction template was transferred into Covidence and used during the final data extraction phase.

The following data were extracted: author, country and year of publication, sample size, population description (age and sex), diagnosis (primary or secondary OA), type of arthroplasty (TKA or UKA), characteristics of physiotherapeutic exercise and control intervention (type, setting, duration, frequency, intensity, start and length of follow-up, supervision), outcome measures, and results in each outcome category (i.e., joint and muscle function, functional performance, and participation). Physiotherapeutic exercise types were divided into these three categories: strengthening exercise (explicitly aimed at improving muscle strength and using external resistance), aerobic exercise, and functional exercise (focused on functional tasks training but not explicitly on strengthening muscles or improving endurance).

### Therapeutic validity

Two researchers independently (AK and SW) assessed the physiotherapeutic exercise interventions’ therapeutic validity utilizing the CONTENT scale for therapeutic validity. The CONTENT scale can be used to assess patient eligibility, competencies and setting, rationale, content, and adherence to the physiotherapeutic intervention. These five critical domains are compromised in a nine-item rating scale. Each question can be answered dichotomously with “yes” (awarded one point) or “no” (awarded no points). A score of 6 or higher indicates good therapeutic validity [18]. Disagreements between the two researchers’ CONTENT scale ratings were solved in a consensus meeting. As the Covidence software does not allow inclusion of a second quality assessment tool, the CONTENT scale was rated outside the software.

### Risk of bias

Risk of bias (RoB) in the findings of the included randomized controlled trials (RCTs) was independently assessed by the same two reviewers (AK and SW) using Cochrane’s Risk of Bias (RoB 2.0) tool. Study quality was assessed using Covidence. To be able to use Covidence during this step, the five RoB 2.0 domains “bias arising from the randomization process”, “bias due to deviations from intended interventions”, “bias due to missing outcome data”, “bias in the measurement of the outcome”, and “bias in the selection of the reported result” were transferred into the customizable quality assessment template within the software. Each bias domain contains signalling questions that can be answered in three ways: “yes/probably yes”, “no/probably no”, and “no information/not applicable”. The answer “not applicable” is only an option for those questions, in which case an earlier question has already provided enough information. The signalling questions led to a certain risk of bias (“low RoB”/ “some concerns”/ “high RoB”) per domain, which is described in Table 1 [28, 29]. Again, any arising conflict was solved in a mutual meeting between the two reviewers.

**Table 1:** Judgement of overall risk of bias RoB 2.0 tool

Overall risk of bias judgement	Criteria
Low risk	The study is judged as “low risk” at all domains
Some concerns	The study is judged as “some concerns” in at least one domain
High risk	The study is judged as “high risk” in at least one domain, or the study is judged to have “some concerns” for multiple domains

## Data synthesis

Articles informing this systematic review contain various physiotherapeutic interventions. Those were evaluated using both objective and subjective/self-reported outcome measures, where within each category several different measurement instruments were used. Because of this heterogeneity it was impossible to perform a meta-analysis. Instead, the Grading of Recommendations, Assessment, Development, and Evaluations (GRADE) method was used to assess the quality of evidence of the included studies. The studies were grouped by outcome measures (joint and muscle function, functional performance, and participation). GRADE was assessed by one reviewer (AK). In case of uncertainty, the other reviewers (GHS or MS) were consulted. The GRADE method consists of three steps: establishing the level of certainty, considering lowering/raising the level of certainty, and assigning the final grade for quality of evidence [30, 31]. Randomized controlled trials are initially assigned a higher grade than observational studies, because they are usually less prone to bias [30]. Reasons to downgrade the level of certainty are: low risk of bias, inconsistency, indirectness of evidence, imprecision, and publication bias [31]. The upgrading criteria are usually applicable for non-randomized studies, but there are exceptions. Reasons to upgrade the level of certainty are: large effect, dose–response relationship, and if all plausible confounders would have reduced the treatment effect. The final GRADE ranking is assigned as *high*, *moderate*, *low*, or *very low* [31].

## Results

### Study selection

**Fig. 1:**
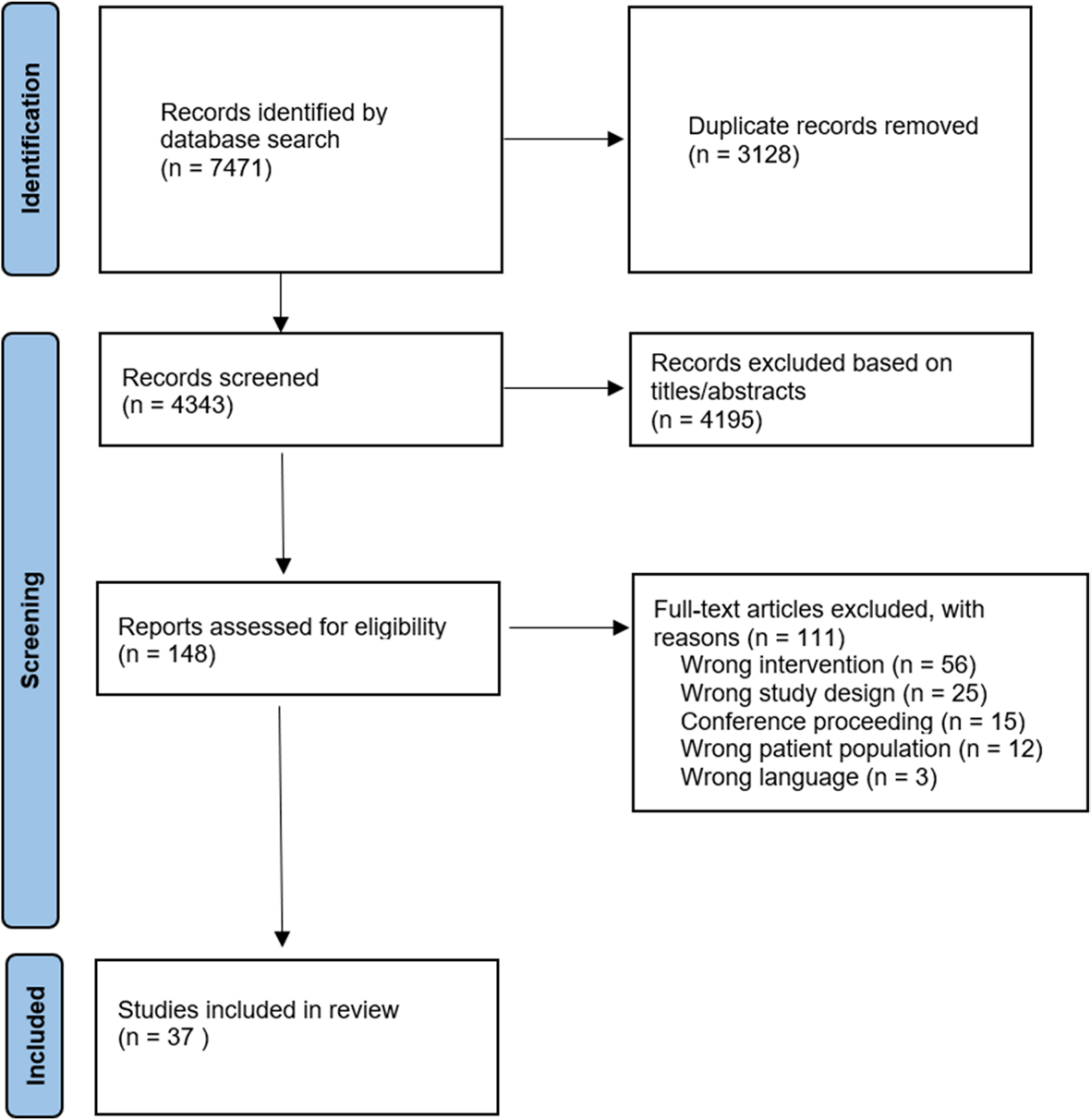
Prisma flow diagram of systematic search, study screening, and selection process

Using the aforementioned search strategy, we identified 7,471 records overall; 3,128 of them were duplicates and were therefore removed. The remaining 4,343 unique records were screened for eligibility based on their title and abstract; 148 of these studies were potentially relevant, therefore the full-text articles were screened. In response to the inclusion and exclusion criteria, 37 studies were ultimately identified as eligible and included in the review. The entire selection process is shown in Fig. 1.

## Study characteristics

Study characteristics of the included articles are shown in Table 2. TKA was assessed in 34 articles [32–65], UKA in one article [66], and two articles included both TKA and UKA [67, 68]. Six studies mentioned that knee arthroplasty was due to primary knee OA [37, 39, 41, 55, 61, 62], the remaining 31 studies gave no OA specifications [32–36, 38, 40, 42–54, 56–60, 62–68]; 13 studies assessed the effect of strengthening exercises [37, 41–43, 45, 46, 48, 51, 52, 60, 62, 65, 66], six focussed on functional exercises [35, 36, 39, 61, 63, 68], and the remaining 18 assessed a combination of these two [32–34, 38, 40, 44, 47, 49, 50, 53–59, 64, 67]. The follow-up periods ranged from four days to twelve months postoperatively, and intervention durations varied between eight days and twelve months.

**Table 2:** Study characteristics of the included articles

	**Participants**					**Intervention group**				**Control group**				**Results**		
**Author (year, country)**	**Study groups: N**	**Age**^**a**^	**Sex (m/f)**	**Type**	**Follow-up**^**b**^	**Type**^**c**^	**Setting**^**d**^** (supervision)**^**e**^	**Duration**^**b**^** (frequency)**^**b**^	**Intensity**^**b,f**^	**Type**^**b**^	**Setting**^**d**^** (supervision)**^**e**^	**Duration**^**b**^** (frequency)**^**b**^	**Intensity**^**b,f**^	**Joint & muscle function**^**g**^	**Functional performance**^**g**^	**Participation**^**g**^
Anneli (2017, Finland) [32]	IG: 53 CG: 55	IG: 69 ± 8 CG: 69 ± 9	IG: 23/30 CG: 19/36	TKA	14 m	F + S	HB; LB (P)	12 m (0-3w: 1x/d; 3w-12 m: 3x/w)	0w-3w: 10–15 reps; 3w-12 m: 15 reps	F + S	H; LB (N)	NR (1-2x/d)	10–15 reps	= /↑	= /↑	=
Bade (2017, US) [33]	IG 84 CG: 78	IG: 63 ± 8 CG: 64 ± 7	IG: 39/45 CG: 34/44	TKA	12 m	F + S	O/HB; LB (P)	11w (0-6w: 3x/w; 7-12w: 2x/w)	2 × 8 reps, 45 min	F + S	O/H; LB (P)	11w (0-6w: 3x/w; 7-12w: 2x/w)	45 min, 2 × 10 reps	=	=	= /↑
Baireddy (2020, India) [34]	IG: 20 CG: 20	IG: 59.1 ± 5.6 CG: 58,1 ± 5.9	NR	TKA	6 m	F + S	O; LB (Y)	NR (NR)	NR	F	O; LB (Y)	NR (NR)	NR	NR	NR	NR
Barker (2020, UK) [67]	IG: 309 CG: 312	IG: 70.67 ± 8.01 CG: 70.18 ± 8.14	IG: 125/184 CG: 125/187	TKA + UKA	12 m	F + S	HB; LB (Y)	NR (7 sessions)	S: 2–3 × 6–12 reps; B: 2–3 × 3 reps	F + S	O; LB (Y)	NR (1–6 sessions)	NR	NA	= /↑	=
Barker (2021, UK) [68]	IG: 309 CG: 312	IG:70.67 ± 8.01 CG:70.18 ± 8.14	IG: 125/184 CG: 125/187	TKA + UKA	12 m	F	HB; LB (P)	NR (NR)	NR	NR	NR (NR)	NR (NR)	NR	= /↓	NA	=
Bruun-Olsen (2013, Norway) [35]	IG: 29 CG: 28	IG: 68 ± 8 CG: 69 ± 10	IG: 11/18 CG: 14/14	TKA	9 m	F	O; LB (Y)	6-8w (12 sessions)	70 min	F + S	O; LB (Y)	6-8w (2x/w)	40 min	=	= /↑	↑
Chow (2010, China) [36]	IG1: 32 IG2: 35 CG: 33	IG1: 67.10 ± 8.02 IG2: 69.95 ± 7.96 CG: 70.10 ± 6.45	IG1: 7/23 IG2: 7/32 CG: 3/36	TKA	2w	F	I; LB (Y)	2w (5x/w)	Stretch 20 s, rest 10 s	F	I; LB (Y)	2w (5x/w)	Stretch 20 s, rest 10 s	=	NA	NA
Codine (2004, France) [37]	IG: 30 CG: 30	IG: 74.6 ± 13 CG: 71.1 ± 15	IG: 14/16 CG: 9/21	TKA	40d	S	I; LB (NR)	3w (5x/w)	15 min	F + S	I; LB (NR)	NR	NR	= /↑	=	NA
Eisermann (2004, Austria) [38]	IG: 75 CG: 72	IG: 70.2 CG:69.7	IG: 23/45 CG: 14/54	TKA	6 m	F + S	I; LB (P)	3-4w (3-5x/w)	30 min	NR	I; LB (Y)	3-4w (3-5x/w)	30 min	=	=	NA
Frost (2002, UK)	IG: 23 CG: 24	IG: 71.5 ± 5.4 CG: 71.1 ± 5.6	IG: 12/11 CG: 12/12	TKA	12 m	F	HB; LB (N)	NR (NR)	NR	F + S	H; LB (N)	NR	NR	=	=	=
Han (2015, Australia) [40]	IG: 194 CG:196	IG: 64.1 ± 6.5 CG:65.4 ± 6.0	IG: 86/108 CG: 92/104	TKA	6w	F + S	HB; LB (N)	6w (3x/d)	10 reps	NR	O; LB (Y)	6w (NR)	NR	=	=	=
Hardt (2018, Germany)	IG: 33 CG: 27	IG:63.3 ± 8 CG:67.6 ± 10.2	IG: 15/18 CG: 11/16	TKA	7d	S	I; LB (N)	7 ± 1d (3-5x/d)	5 min	F	I; LB (Y)	7 ± 1d (NR)	NR	= /↑	= /↑	= /↑
Hsu (2019, China) [42]	IG: 20 CG: 20	IG:72.0 ± 1.8 CG: 69.5 ± 1.5	NR	TKA	36w	S	O; LB (Y)	24w (3x/w)	3 × 12 reps + 1-4w: 60% 1RM; 5-8w: 70% 1 RM; 9-24w: 12 m 80% 1RM	F + S	H; LB (N)	NR (NR)	NR	=	=	= /↑
Husby (2018, Norway) [43]	IG: 21 CG: 20	IG: 61 CG: 63	IG: 10/11 CG: 8/12	TKA	12 m	S	O/HB; LB (P)	8w (3x/w)	4 × 5 reps,with load 80–90% of 1 RM, 30 min	NR	O/H; LB (P)	8d-10w (NR)	NR	= /↑	↑	=
Jacksteit (2021, Germany) [44]	IG_uni:22 IG_bi:22 CG: 22	IG_uni:67.36 ± 9.61 IG_bi:68.55 ± 8.94 CG: 68.45 ± 9.07	IG_uni: 9/13 IG_bi: 8/14 CG: 8/14	TKA	3 m	F + S	I; LB (Y)	8d (IG_uni: 3x/d; IG_bi: 3x/d affected + 1x/d healthy)	30 min	F	I; LB (Y)	8d (3x/d)	30 min	=	=	= /↑
Jørgensen (2017, Denmark) [66]	IG: 31 CG: 24	IG: 64.8 ± 8.3 CG: 64.4 ± 8.7	IG:16/15 CG:10/14	UKA	12 m	S	O/HB; LB (P)	8w (2x/w + 5x/w)	2–4 × 8-12RM	F	H;LB (P)	8w (3x/w)	10 reps	=	=	NR
Karaman (2017, Turkey) [45]	IG: 23 CG: 23	IG: 67.6 ± 7.0 CG: 70.1 ± 6.9	NR	TKA	6w	S	HB; LB (Y)	6w (NR)	NR	S	I/H; LB (Y)	6w (NR)	Isometric: 10 reps/h; isotonic: 3 × pd 5 reps	NA	↑	↑
Lenguerrand (2020, UK) [46]	IG: 89 CG: 91	IG: 69 ± 9 CG: 69 ± 9	IG: 39/50 CG: 42/49	TKA	12 m	S	O; LB (Y)	6w (1x/w)	1 h	F	O/H; LB (Y)	6w (NR)	NR	= /↑	NA	NA
Lenssen (2006, Netherlands) [47]	IG: 21 CG: 22	IG: 70 ± 8.5 CG: 67 ± 7	IG: 6/15 CG: 5/17	TKA	3 m	F + S	I; LB (Y)	4d (2x/d)	20 min	F + S	I; LB (Y)	4d (1x/d)	20 min	=	=	=
Li (2019, China) [52]	IG: 54 CG: 53	IG: 69.6 ± 4.3 CG: 68.5 ± 3.5	IG:26/28 CG:24/29	TKA	14w	S	HB; LB (P)	12w (5 × pw)	45 min	S	LB (NR)	12w (5x/w)	45 min	=	↑	= /↑
Liao (2013, Taiwan) [50]	IG: 65 CG: 65	IG: 71.38 ± 6.57 CG: 72.94 ± 7.33	IG: 18/37 CG: 12/46	TKA	8w	F + B	O; LB (P)	8w (NR)	60 min	F	O; LB (P)	8w (NR)	90 min	NA	↑	↑
Liao (2015, Taiwan)	IG: 65 CG: 65	IG:71.43 ± 6.33 CG: 73.40 ± 7.04	IG: 16/49 CG: 23/42	TKA	32w	B	LB (P)	8w (3x/w)	NR	F	I; LB (Y)	8w (3x/w)	NR	NA	↑	↑
Liao, Chiu (2020, Taiwan) [48]	IG: 20 CG: 20	IG:72.22 ± 7.75 CG: 69.79 ± 6.72	NR	TKA	16w	S	HB; LB (P)	3 m (2x/w)	3 × 10–20 reps, 55 min	F	O; LB (NR)	NR (NR)	NR	NR	↑	=
Liao, Tsauo (2020, Taiwan) [51]	IG: 30 CG: 30	IG: 72.22 ± 7.75 CG: 69.79 ± 6.72	IG: 0/30 CG: 0/30	TKA	4 m	S	HB; LB (P)	4 m (2x/w)	3 × 10–20 reps, 60 min	F	O; LB (Y)	4 m (2x/w)	60 min	NA	= /↓/↑	↑
McAvoy (2009, UK) [53]	IG: 15 CG:15	NR	IG:7/8 CG: 6/6	TKA	6 m	F + S	O; LB/WB (Y)	6w (2x/w)	60 min	F + S	O;LB (Y)	6w (2x/w)	60 min	= /↑	NA	=
Mockford (2008, UK) [54]	IG: 71 CG: 72	IG: 69.4 CG: 70.9	IG: 25/46 CG:30/42	TKA	12 m	F + S	O/HB; LB (Y)	NR (9 sessions)	NR	NR	H; LB(Y)	NR	NR	=	NA	=
Moffet (2004, Canada) [55]	IG: 38 CG: 39	IG: 66.7 ± 8.7 CG: 86.7 ± 8.3	IG: 14/24 CG: 17/22	TKA	8 m	F + S + A	O/HB; LB (P)	6-8w (12 sessions)	60–90 min; 8–25 reps per modality	NR	H; LB (P)	2-4 m (7–10 visits)	NR	NA	= /↑	= /↑
Piva (2010, US) [56]	IG: 21 CG: 22	IG: 67 ± 6 CG: 70 ± 10	IG: 13/5 CG: 12/5	TKA	6 m	F + S + A	O/HB; LB (Y)	6w (2x/w)	10–20 reps	F + S + A	O/H; LB (Y)	6w (2x/w)	10–20 reps	NA	=	=
Rahmann (2009, Australia) [57]	IG1: 24 IG2: 21 CG: 20	IG1: 69.4 ± 6.5 IG2: 69.0 ± 8.9 CG: 70.4 ± 9.2	IG1: 5/12 IG2: 10/11 CG: 7/12	TKA	180d	F + S	I; LB/WB (Y)	NR (1x/d)	40 min, 10–30% WB	F + S	I; LB (Y)	NR (1x/d)	40 min	NA	= /↑	=
Roig-Casasus (2018, Spain) [58]	IG: 19 CG: 24	IG: 74.8 ± 4.0 CG: 72.1 ± 4.5	IG: 5/12 CG: 7/13	TKA	10w	F + S	O;LB (Y)	4w (5x/w)	80 min	F + S	O; LB (Y)	4w (5x/w)	60 min	NA	= /↑	NA
Sano (2018, Japan) [59]	IG: 37 CG:38	IG: 75.0 ± 6.4 CG:75.8 ± 5.8	IG: 7/30 CG:8/30	TKA	3w	F + S	I; LB (Y)	3w (5x/w)	5 reps	F + S	I;LB (Y)	3w (5x/w)	5 reps	=	↑	=
Schache (2019, Australia) [60]	IG: 54 CG: 51	IG: 70 ± 7 CG: 69 ± 7	IG: 15/39 CG: 21/30	TKA	26w	S	I; LB (Y)	6w (5x/w)	60 min	F	I; LB (Y)	6w (5x/w)	60 min	=	=	= /↑
Shabbir (2017, Pakistan) [61]	IG: 31 CG: 33	IG: 60 ± 10 CG: 72.1 ± 6.3	NR	TKA	4w	F	HB; LB (Y)	NR (NR)	NR	S	O; LB (Y)	NR (1x/d, 5x/w)	NR	=	NA	=
Teissier (2020, France) [62]	IG: 10 CG: 10	IG + CG: (72.1 ± 6.3)	8/12	TKA	4w	S	I; LB (Y)	4w (10x/w, 2x/d)	3–4 × 5–10 reps	F	O; LB (NR)	NR (NR)	3–5 × 5–10 reps	=	↑	=
Torpil (2022, Turkey) [63]	IG: 21 CG: 20	IG: 55.10 ± 5.95 CG: 55.31 ± 5.45	IG: 4/15 CG: 5/14	TKA	12d	F	HB; LB (Y)	12d (1x/d)	45 min	NR	NR (NR)	NR (NR)	NR	NA	NA	=
Vuorenmaa (2014, Finland) [64]	IG: 53 CG: 55	IG: 69 ± 8 CG: 69 ± 9	IG: 30/23 CG: 36/19	TKA	12 m	F + S	HB; LB (P)	12 m (3x/w)	2–3 × 10–20 reps	NR	NR (NR)	NR (NR)	NR	= /↑	= /↑	=
Warner (2020, Pakistan) [65]	IG: 22 CG: 22	IG + CG: 62.93 ± 9.146	15/29	TKA	6w	S	I; LB (Y)	6w (6x/w)	10 × 2 reps	S	LB (Y)	6w (NR)	NR	NA	↑	NA

*IG* intervention group, *CG* control group, *NR* not reported, *NA* not applicable

^a^Age (years) is shown as mean ± SD or only mean

^b^d = days; w = weeks; m = months; y = year

^c^Type: F = functional; S = strengthening; B = balance; A = aerobic/endurance

^d^Setting: I = inpatient; O = outpatient; HB = home-based; LB = land-based; WB = water-based

^e^Supervision: Y = yes; P = partially; N = no

^f^Intensity: min = minutes; h = hour; reps = repetitions; RM = repetition maximum

^g^Effect of intervention: ↑ significant difference favouring IG; ↓ significant difference favouring CG; = no significant difference between IG and CG; a combination means that more results were found

## Therapeutic validity

Table 3 shows the results obtained for the therapeutic validity assessment. Absolute agreement between both reviewers’ ratings was achieved in 80.8% of cases (269 out of 333 items). Overall, the total scores of the studies ranged from zero to seven points. Only six (16%) out of the 37 studies informing this review [33, 55, 63, 64, 67, 68] scored six points or higher on the CONTENT scale, suggesting low therapeutic validity in 31 studies. Exercise personalization (content domain) scored “yes” the least often; only three articles (8%) [38, 48, 63] addressed this item. The item adequate patient eligibility achieved the highest total score, as it was reported in 31 studies (89%) [32–37, 39, 40, 42–48, 50–52, 55–68].

**Table 3:** Results of the CONTENT scale for each included article

Study	Patient eligibility		Competence & setting	Rationale		Content			Adherence	Total score
	**Described**	**Adequate**		**Intentions & hypothesis**	**Content**	**Intensity**	**Monitored**	**Personalized**		
Anneli (2017) [32]	yes	yes	no	no	no	yes	no	no	no	3
Bade (2017) [33]	yes	yes	yes	yes	no	yes	yes	no	yes	**7**
Baireddy (2020) [34]	yes	yes	no	yes	no	no	no	no	no	3
Barker (2020) [67]	yes	yes	no	yes	no	yes	yes	no	yes	**6**
Barker (2021) [68]	yes	yes	yes	yes	no	no	yes	no	yes	**6**
Bruun-Olsen (2013) [35]	yes	yes	no	no	no	no	yes	no	no	3
Chow (2010) [36]	yes	yes	no	no	no	no	no	no	no	2
Codine (2004) [37]	no	yes	no	no	no	no	no	no	no	1
Eisermann (2004) [38]	no	no	no	no	yes	no	yes	yes	no	3
Frost (2002) [39]	yes	yes	no	no	no	no	no	no	no	2
Han (2015) [40]	yes	yes	no	no	no	no	yes	no	no	3
Hardt (2018) [41]	no	no	no	yes	no	no	no	no	no	1
Hsu (2019) [42]	no	yes	no	no	no	yes	yes	no	no	3
Husby (2018) [43]	yes	yes	no	yes	no	yes	yes	no	no	5
Jacksteit (2021) [44]	yes	yes	no	yes	no	yes	yes	no	no	5
Jørgensen (2017) [66]	yes	yes	no	yes	no	yes	yes	no	no	5
Karaman (2017) [45]	yes	yes	no	yes	no	no	no	no	no	3
Lenguerrand (2020) [46]	no	yes	no	no	no	no	no	no	yes	2
Lenssen (2006) [47]	no	yes	no	no	no	no	no	no	no	1
Liao (2013) [50]	yes	yes	no	yes	no	no	no	no	no	3
Liao (2015) [49]	no	no	no	yes	no	no	no	no	yes	2
Liao, Chiu (2020) [48]	yes	yes	no	no	yes	yes	no	yes	no	5
Liao, Tsauo (2020) [51]	yes	yes	no	no	no	yes	yes	no	yes	5
Li (2019) [52]	yes	yes	yes	no	no	yes	no	no	no	4
McAvoy (2009) [53]	yes	yes	no	yes	no	no	no	no	no	3
Mockford (2008) [54]	no	no	no	no	no	no	no	no	no	0
Moffet (2004) [55]	yes	yes	no	no	yes	yes	yes	no	yes	**6**
Piva (2010) [56]	yes	yes	no	no	yes	yes	no	no	yes	5
Rahmann (2009) [57]	yes	yes	no	no	no	yes	yes	no	no	4
Roig-Casasus (2018) [58]	yes	yes	no	no	no	yes	yes	no	yes	5
Sano (2018) [59]	yes	yes	no	yes	no	yes	yes	no	no	5
Schache (2019) [60]	yes	yes	no	no	no	no	no	no	no	2
Shabbir (2017) [61]	no	yes	no	no	no	no	no	no	no	1
Teissier (2020) [62]	yes	yes	no	no	yes	yes	yes	no	no	5
Torpil (2022) [63]	yes	yes	yes	no	yes	no	yes	yes	no	**6**
Vuorenmaa (2014) [64]	yes	yes	no	yes	no	yes	yes	no	yes	**6**
Warner (2020) [65]	no	yes	no	no	no	yes	no	no	no	2
Total “yes”	27 (73%)	33 (89%)	4 (11%)	14 (38%)	6 (16%)	18 (49%)	18 (49%)	3 (8%)	10 (27%)	

## Risk of bias

Table 4 shows the results of the risk of bias rating. An absolute between-rater agreement was achieved in 126 out of 185 items (68.1%). The domain “selection of reported result” scored best, with 32 articles (92%) scoring “low RoB” [32–36, 38–47, 49–60, 63, 64, 66–68]. By contrast, the domain “deviations from the intended process” scored as “low RoB” in only 24% of the studies [41, 44, 51, 55, 56, 61, 62, 65, 68]. In total, three articles (8%) [51, 56, 68] achieved an overall low RoB score. Fifteen studies (41%) scored “some concerns for RoB” [33, 34, 39–41, 43–45, 47, 49, 50, 55, 57, 58, 60, 64] and the other 19 studies (51%) scored “high RoB” [32, 35–38, 42, 46, 48, 52–54, 59, 61–63, 65–67].

**Table 4:** Results of RoB 2.0 tool for each included article

**Study**	Randomization process	Deviations from the intended process	Missing outcome data	Measurement of the outcome	Selection of reported result	Total
Anneli (2017) [32]	Low	High	Low	Low	Low	High
Bade (2017) [33]	Low	Some concerns	Low	Low	Low	Some concerns
Baireddy (2020) [34]	High	Some concerns	Low	Low	Low	Some concerns
Barker (2020) [67]	Low	High	Low	Low	Low	High
Barker (2021) [68]	Low	Low	Low	Low	Low	**Low**
Bruun-Olsen (2013) [35]	Low	High	Low	Low	Low	High
Chow (2010) [36]	Low	Some concerns	Low	Some concerns	Low	High
Codine (2004) [37]	Low	High	Some concerns	Some concerns	Some concerns	High
Eisermann (2004) [38]	Some concerns	Some concerns	Low	High	Low	High
Frost (2002) [39]	Low	Some concerns	Low	Low	Low	Some concerns
Han (2015) [40]	Low	Some concerns	Low	Low	Low	Some concerns
Hardt (2018) [41]	Low	Low	Some concerns	Low	Low	Some concerns
Hsu (2019) [42]	High	High	High	High	Low	High
Husby (2018) [43]	Low	Some concerns	Low	Some concerns	Low	High
Jacksteit (2021) [44]	Low	Low	Low	Some concerns	Low	Some concerns
Jørgensen (2017) [66]	Low	Some concerns	Some concerns	Low	Low	High
Karaman (2017) [45]	Low	Some concerns	Low	Low	Low	Some concerns
Lenguerrand (2020) [46]	Low	Some concerns	Low	High	Low	High
Lenssen (2006) [47]	Low	Some concerns	Low	Low	Low	Some concerns
Liao (2013) [50]	Low	Some concerns	Low	Low	Low	Some concerns
Liao (2015) [49]	Low	Some concerns	Low	Low	Low	Some concerns
Liao, Chiu (2020) [48]	Low	Some concerns	Low	Some concerns	Some concerns	High
Liao, Tsauo (2020) [51]	Low	Low	Low	Low	Low	**Low**
Li (2019) [52]	Low	Some concerns	Low	Some concerns	Low	High
McAvoy (2009) [53]	High	Some concerns	Some concerns	Low	Low	High
Mockford (2008) [54]	Low	Some concerns	Low	High	Low	High
Moffet (2004) [55]	Low	Low	Low	Some concerns	Low	Some concerns
Piva (2010) [56]	Low	Low	Low	Low	Low	**Low**
Rahmann (2009) [57]	Low	Some concerns	Low	Low	Low	Some concerns
Roig-Casasus (2018) [58]	Low	Some concerns	Low	Low	Low	Some concerns
Sano (2018) [59]	Low	Some concerns	Low	Some concerns	Low	High
Schache (2019) [60]	Low	Some concerns	Low	Low	Low	Some concerns
Shabbir (2017) [61]	Low	Low	Low	Low	High	High
Teissier (2020) [62]	Some concerns	Low	Low	Some concerns	High	High
Torpil (2022) [63]	High	Some concerns	Low	High	Low	High
Vuorenmaa (2014) [64]	Low	Some concerns	Low	Low	Low	Some concerns
Warner (2020) [65]	Low	Low	High	Some concerns	High	High
Total low (%)	31 (84%)	9 (24%)	31 (84%)	22 (59%)	32 (86%)	3 (8%)

### Therapeutic validity and Risk of Bias

Five of the six articles that met the criteria for sufficient therapeutic validity did not meet the criteria for low risk of bias [33, 55, 63, 64, 67]. Two articles met the criteria for low risk of bias but with a score of less than six points on the CONTENT scale missed the criteria for high therapeutic validity [48, 56]. Only one article scored sufficiently on therapeutic validity and risk of bias [68].

## Characteristics and effectiveness of the physiotherapeutic exercise interventions

Table 2 shows the results of the included articles’ physiotherapeutic interventions. Again, there was a large variability between studies in physiotherapeutic exercise interventions used and type of outcome measures applied.

### Outcome measures

Based on the ICF scheme, the study results were grouped into three different categories: joint and muscle function, functional performance, and participation, with results for each category reported in 68%, 78%, and 78% of the studies, respectively. Different outcome measures and units of measurement were used within categories.

Joint and muscle functions were primarily measured in terms of ROM and strength. Joint ROM was reported in degrees and measured during both active and passive movement in 20 articles [32, 34–41, 43, 44, 47, 48, 52–54, 59, 60, 62, 64]. Hip abductor [34, 57, 60], quadriceps muscle [33, 57, 59–61], and hamstrings [33, 57, 59] strength were evaluated in three, five, and three articles, respectively. Strength values were measured in these units of measurement: pounds, kilograms, Newton normalized to BMI, and Newton-metres/kilograms. One article used the Index of Muscle Function (IMF) test to perform stress testing [35]. In 64% of the articles reporting on the outcome measure joint and muscle function, there was no statistically significant difference between IG and CG [33, 35–40, 42, 44, 47, 52, 54, 59–62, 66].

Functional performance measurements have been executed in various ways. A total of 17 measurements were used to obtain the intended results in the different articles. The 6-min walk test (6MWT) [33–35, 42, 43, 52, 55, 60] and the Timed Up and Go test (TUG) [33, 41, 44, 51, 58–60, 62, 64] were used in most studies to measure functional performance. Other studies measured and reported functional performance using subjects’ maximal walking speed [38, 39, 49, 51, 59, 64], the stair-climbing test [33, 35, 42, 44, 49, 60, 67], single-leg stance test [38, 49–51, 56, 60, 67, 68], figure-of-eight test [35, 42, 67, 68], functional reach test [58, 59], 10-min walk test [41, 50, 62], 30-s chair test [42, 44, 60], Berg balance scale [45, 58], and subjects’ step cadence [49, 66]. The 50-foot walking test was reported in one article [36].

For participation, 15 disease-specific and generic measuring instruments were used throughout the studies. The most commonly used disease-specific questionnaire was the Western Ontario and McMaster Universities Osteoarthritis Index (WOMAC), in 10 studies [33, 40, 47–52, 55–57, 62, 64]. Other disease-specific tools included the Knee Injury and Osteoarthritis Outcome Score (KOOS) [35, 41–43, 46, 60, 68] and the knee society score (KSS) [41]. The following generic measuring instruments were also used frequently: Short Form Health Survey 12 (SF-12) [33, 54, 60], Late-Life Function & Disability Instrument (Late-Life FDI) [67, 68], 5-level EQ-5D [67, 68], Oxford knee score (OKS) [54, 67, 68], Physical Activity Scale for the Elderly (PASE) [67, 68], Short Form Health Survey 36 (SF-36) [43, 44, 52], and Lower Extremity Functional Scale (LFSE) [60, 65]. The Functional Independence Measure (FIM) [34], sleep efficiency score [35], KSS [41], and Canadian Occupational Performance Measure (COPM) [63] were reported in one article each.

### Interventions

Seventeen studies (46%) [32–39, 41, 43, 44, 47, 50, 56, 58–60] applied the intervention in the same setting for both IG and CG, while the setting for IG and CG interventions was different in 14 studies (38%) [40, 42, 45, 46, 48, 51, 53–55, 57, 61, 62, 66, 67]. Six studies (16%) did not report on the exercise intervention setting at all [49, 52, 63–65, 68]. The IG intervention was water-based in only two studies (5%) [53, 57]. The majority (32%) of the physiotherapeutic exercises the IG received were land-based and performed in the patient’s home environment [32, 39, 40, 45, 48, 51, 52, 61, 63, 64, 67, 68]. The physiotherapeutic exercises given to the IG were mostly inpatient (30%) [36–38, 41, 44, 47, 57, 59, 60, 62, 65], and to a lesser amount outpatient (19%) [34, 35, 42, 46, 50, 53, 58]. In 16% of the studies, the intervention received was both outpatient and home-based [33, 43, 54–56, 66]. Also, 3% did not report the setting of the given physiotherapeutic exercises for the IG [49]. The CGs received their interventions mostly in outpatient settings (30%) [34, 35, 40, 48, 50, 51, 53, 58, 61, 62, 67], followed by inpatient settings (27%) [36–38, 41, 44, 47, 49, 57, 59, 60] and home-based settings (16%) [32, 39, 42, 54, 55, 66]. In 11% of the articles, the CG received both outpatient and home-based interventions [33, 43, 46, 56], while in 3% of studies a combination of inpatient and home-based interventions [45] was used. The care setting of the CG was not stated in 15% of the articles [52, 63–65, 68]. While in 46% of the studies there was supervision during the entire intervention for both IG and CG [34, 35, 44–47, 53, 54, 56–61, 65, 67], in 14% of the studies there was only partial supervision for both groups [33, 43, 50, 55, 66]. In 3% of the studies, there was no supervision either group [39]. In the remaining cases, supervision during the exercise interventions was either not reported or varied between IG and CG [32, 36–42, 48, 49, 51, 52, 62–64, 68].

Twenty-three studies used functional exercises (e.g., Sit-to-Stand and other transfer exercise, walking exercises) [32–36, 38–40, 44, 47, 50, 53–59, 61, 63, 64, 67, 68], while 29 investigated the effect of strengthening (e.g., leg press, quadriceps curl, and hamstrings curl) [32–34, 37, 38, 40–48, 51–60, 62, 64, 66, 67]. Two articles studied balance exercises (e.g., single leg balance exercises) [49, 50] and two studies aerobic/endurance exercises (e.g., ergometer, cross-trainer) [55, 56]. The CG got usual care in 17 studies [32, 34, 35, 38, 40, 41, 43, 45–48, 51, 52, 55, 59, 67, 68]. The exact intervention for the CG was not reported in 20 studies [33, 36, 37, 39, 42, 44, 49, 50, 53, 54, 56–58, 60–66]. Length of follow-up varied from seven days to 14 months.

#### GRADE

Certainty of evidence was assessed using the most-often used outcome measure per outcome category. Knee ROM, TUG, and WOMAC representing the categories joint and muscle function, functional performance, and participation, respectively, were evaluated because these outcome measures were most commonly performed in the different studies informing this review. Most studies lacked blinding, explanations for heterogeneity of results, inconsistency, homogeneity of exercises between studies, and inaccuracy. Quality was therefore downgraded for every outcome category, while upgrading was not applicable. For this reason, the quality of evidence for all three outcomes had to be classified as *very low* (Table 5).

**Table 5:** Certainty of evidence per outcome category (GRADE)

Study	Groups		Difference between groups	GRADE
	IG	CG	IG-CG	
Joint and muscle function (Knee ROM, flexion, in °)				Very Low
Anneli (2017) [32]	150	120	30 (+ 25%)	
Bade (2017) [33]	126	126	0 (0%)	
Codine (2004)^a^ [37]	22.32	6.25	16.07 (+ 257%)	
Eisermann (2004)^a^ [38]	10.4	9.4	1(+ 10%)	
Frost (2002) [39]	102	102	0 (0%)	
Han (2015) [40]	96.8	95.7	1.1 (+ 1%)	
Jacksteit (2021) [44]	113.77	110.33	3.44 (+ 3%)	
Liao, Chiu (2020)^a^ [48]	28.08	20.58	7.5 (+ 36%)	
Li (2019) [52]	112.1	110	2.1 (+ 2%)	
Mockford (2008) [54]	107.9	106.6	1.3 (+ 1%)	
Sano (2018) [59]	119.1	121.1	2 (+ 2%)	
Schache (2019) [60]	120	117	3 (3%)	
Teissier (2020) [62]	18	10	8 (80%)	
Vuorenmaa (2014) [64]	14.4	14.2	0.2 (+ 1%)	
Functional performance (TUG)^b^				Very low
Bade (2017) [33]	17.20	16.41	0.79 (+ 5%)	
Hardt (2018) [41]	12.1	17.8	-5.7 (-32%)	
Jacksteit (2021) [44]	8.82	10.18	-1.36 (-13%)	
Liao, Tsauo (2020) [51]	9.13	12.32	-3.19 (-26%)	
Roig-Casasús (2018) [58]	14.4	17.3	-2.9 (-17%)	
Sano (2018) [59]	10.44	12.22	-1.78 (-15%)	
Schache (2019) [60]	8	8	0 (0%)	
Vuorenmaa (2014) [64]	-1.58	-0.43	-1.15 (-73%)	
Participation (WOMAC, pain)^b^				Very low
Han (2015) [40]	7.2	7.4	-0.2 (-3%)	
Lenssen (2006) [47]	9.2	8.3	0.9 (+ 10%)	
Liao (2015) [49]	22.4	22.4	0 (0%)	
Liao, Chiu (2020)^a^ [48]	-6.95	-5.55	1.4 (20%)	
Liao, Tsauo (2020) [51]	9.1	9.3	-0.2 (-2%)	
Li (2019) [52]	9.1	9.3	-0.2 (-2%)	
Moffet (2004) [55]	9.4	11.8	-2.4 (-26%)	
Piva (2010)^a^ [56]	-30	-27	3 (+ 10%)	
Vuorenmaa (2014)^a^ [64]	-15	-14	1 (+ 7%)	

^a^Not the total score, but the difference with the baseline score

^b^A lower score indicates a better result

## Discussion

The main goal of the present systematic review was to determine the effectiveness and the therapeutic validity of physiotherapeutic exercise in the domains of joint and muscle function, functional performance, and participation following TKA and/or UKA for OA. It was hypothesized that higher therapeutic validity of the applied physiotherapeutic exercises would be more effective and thus result in superior recovery after TKA/UKA. However, our findings indicate that the therapeutic validity of the included articles was mostly insufficient, as only the exercise interventions from six out of the 37 studies could be rated as having high therapeutic validity. The methodological quality of the articles that inform this systematic review was insufficient – only three of the 37 papers presented an overall low risk of bias. Interestingly, only one article [68] scored well on both methodological quality and therapeutic validity. In summary, heterogeneity of outcome measures and length of follow-up, limited reporting of details of the physiotherapeutic exercises and control interventions, and low methodological quality of most studies render an accurate evaluation of the effectiveness of physiotherapeutic exercises after TKA/UKA impossible. Hence based on our findings, the hypothesis that a higher therapeutic validity of the applied physiotherapeutic exercises would be more effective, resulting in superior recovery after TKA/UKA, cannot yet be confirmed.

## Therapeutic validity

Only six out of 37 (16%) studies scored sufficiently for therapeutic validity. Although this may indeed be the result of an insufficient exercise programme, it could also be due simply to a lack of sufficiently detailed description and reporting of the physiotherapeutic interventions. The fact that the CONTENT scale domains may not have been described does not necessarily mean they haven’t been applied in the study. Still, enough of a description is necessary to enable researchers and clinicians to arrive to a valid conclusion about the physiotherapeutic intervention’s quality and effectiveness [19]. We therefore echo Bandholm and Kehlet [69] plus several other authors [18, 21–24] who have emphasized the importance of always determining the therapeutic validity in RCTs of physiotherapeutic exercise intervention studies besides the methodological risk of bias.

The CONTENT scale item “was the therapeutic exercise personalized and contextualized to the individual participants?” was the least reported in the articles informing the current systematic review. This item aims not only to determine whether the therapeutic exercises’ goals and content match patients’ bodily functions and structures, activities, and participation levels but also their personal and environmental factors [18]. In terms of personal factors, aspects such as motivation, coping, and ethnicity could affect the outcome [18]. Environmental factors include logistics, support of family and friends, and technology (i.e., mobile phone). For example, five articles mentioned a maximum travel distance or the need for a supportive partner in the environment but did not consider any personal factors [32, 35, 43, 52, 66]. Previous research shows that motivation can be an essential factor in the rehabilitation process and is related to rehabilitation outcomes [70]. The ICF model offers help to describe these factors in an article [11].

The CONTENT scale has been used in a few other articles before, and their results align on therapeutic validity with those of the current review. The most recent paper is by Wijnen et al. (2018), who examined whether the therapeutic validity of exercise interventions was related to physiotherapeutic exercises following total hip arthroplasty [24]. They included 20 articles, only one of them showing high therapeutic validity. Vooijs et al. (2015) investigated the effectiveness of exercise training in patients with chronic obstructive pulmonary disease [21]. Of the 13 studies included, six were assessed as having high therapeutic validity. However, they found no significant effect between therapeutic validity and physical exercise employing a meta-analysis. Snoek et al. (2013) investigated the relation between aerobic training and heart rate recovery in patients with established heart disease [23]. They included eight studies, three with good therapeutic validity. Last, Hoogeboom et al. (2012) investigated the effect of preoperative exercise on functional recovery after total joint replacement [18]. None of their twelve included studies met therapeutic validity. It emerged that none of the therapeutic exercises showed a significant effect on functional recovery. However, the poor therapeutic validity may have adversely affected the outcomes, as none of the studies met the predefined criteria. Overall, it can be concluded that the results of the present review are in line with the previous articles. Consequently, the potential effectiveness of the physiotherapeutic exercise interventions is impossible to estimate. However, whether the CONTENT scale aspects were not applied during the development of the intervention protocols or whether these aspects were just not reported in the articles remains an open question.

## Methodological quality

Methodological quality revealed only three of the 37 articles as having low risk of bias as determined with the RoB 2.0 tool. The domain “bias due to deviations from intended interventions” scored “high risk of bias” most often compared to the other RoB 2.0 domains. This is mostly because the participants and/or caregivers were not blinded to the given intervention. Research has shown that blinding subjects and/or researchers is important because knowledge about the intervention can potentially influence a study’s results [71]. For example, participants may be excited and hopeful when they receive a new treatment and/or are allocated to the treatment group, yet may be disappointed and thus less motivated when they receive regular treatment or a placebo [71]. As blinding participants in studies using physiotherapeutic exercises is hard to achieve because they can see what kind of exercises they have to do, what can and should be done alternatively to increase the methodological quality is blinding the researchers who do the measurements. Most articles did not perform or report an a priori sample size calculation [32–35, 40, 42, 45, 48, 51, 58–61, 64]. Those studies may be underpowered and therefore have a greater chance of a type II error.

 A previous review shows that the RoB 2.0 tool is not always used according to regulations, as using this tool is challenging [72]; such challenges may allow for low inter-rater reliability to emerge [73]. The current review also shows that the untrained reviewers had lower inter-rater reliability when using the RoB 2.0 tool versus other rating instruments, such as the CONTENT scale (68.1% vs. 80.8%, respectively). More intensive training and developers’ guidance in using the RoB 2.0 tool may significantly improve the reliability of this instrument [74].

## Effectiveness

The results of the current systematic review show significant heterogeneity among physiotherapeutic interventions following TKA/UKA. This is due to a great variation in studied sample size, follow-up periods, intervention settings, exercise supervision, type of intervention, duration and frequency, and intensity for both the physiotherapeutic exercise interventions and the control interventions. Given the diversity in outcome measures used to evaluate the effect of exercises, this precludes a clear answer as to the extent to which joint and muscle function, functional performance, and participation following TKA or UKA for OA could improve with physiotherapeutic exercises. In addition, according to the GRADE approach the certainty of evidence is very low. This finding emphasizes the inadequacy of available studies in this area of research.

## Strengths and limitations

The present review presents both strengths and limitations. To our knowledge, this is the first systematic review to assess the therapeutic validity of physiotherapeutic interventions after TKA/UKA based on the CONTENT scale. We strictly followed the PRISMA guidelines. The search strategy was developed in cooperation with an experienced scientific librarian, and multiple relevant databases were searched. The screening process and the custom-built data extraction sheet’s usefulness were piloted, with good results. Another strength is that GRADE has been used to interpret the strength of the results. Last, we only included studies that concentrated on patients who received a TKA or UKA due to OA and excluded articles on TKA/UKA indications other than OA, such as fractures or infections.

A limitation of this systematic review is that only one of the included articles investigated UKA, while the rest were about TKA. Although we followed a comprehensive search strategy, relevant articles might have been missed due to language or publication period restrictions. In addition, we did not contact the authors of one study, which did not have the results mentioned in their article, to learn more about their findings. Last, study selection criteria were developed by the reviewers themselves, which could have led to selection bias of included studies.

## Future research

For future research, it is advised for studies involving physiotherapeutic exercise interventions that both aspects of therapeutic and methodological validity be used in the development and reporting of the studies. Adding the therapeutic validity provides more clarity about the physiotherapeutic intervention used. The more in-depth information on the used therapeutic exercise that is reported, the better the effects of the physiotherapeutic intervention can be assessed. In addition, given the diversity in outcome measures reported it is advised to use an endorsed set of outcome measures in future investigations, which at this moment does not exist for therapeutic studies. However, such a core outcome set could be developed by example of the Outcome Measures in Rheumatology (OMERACT) initiative’s total joint replacement core domains [75]. Furthermore, more well-designed RCTs that examine the effects of physiotherapeutic exercise interventions on joint and muscle function, functional performance, and participation outcomes following TKA and UKA are warranted to provide added homogeneous evidence. Only with more high-quality evidence will researchers and clinicians be able to understand and scoop the use of physiotherapeutic exercises for their TKA/UKA patients.

## Conclusion

The combination of insufficient therapeutic validity of physiotherapeutic exercises and low methodological quality of the included studies precludes conclusive inferences about the effectiveness of physiotherapeutic exercise interventions on joint and muscle function, functional performance, and participation following TKA/UKA for OA. To be able to draw distinct conclusions, use of physiotherapeutic exercise interventions in this patient population is worth further investigation. The methodological shortcomings in the available studies – such as small sample sizes, lack of blinding, possible insufficient reporting, and heterogenous outcome measures – could be overcome by larger-scale, prospective, long-term follow-up studies utilizing a standardized core outcome set including objective and self-reported outcomes in patients with TKA/UKA, and use of the CONTENT scale as a template to prevent insufficient study design and reporting.

## Supplementary Information

**Additional file 1**. S2 Search strategy.

## Data Availability

The datasets used and/or analysed for the current study are available from the corresponding author upon reasonable request.

## Abbreviations

UKA: Unicompartmental knee arthroplasty
TKA: Total knee arthroplasty
CONTENT scale: Consensus on Therapeutic Exercise Training scale
OA: Osteoarthritis
QoL: Quality of Life
ICF: International Classification of Functioning, Disability and Health
PRISMA: Preferred Reporting Items for Systematic Reviews and Meta-Analyses
PROSPERO: International prospective register of systematic reviews
PICOS: Patient, Intervention, Comparisons, Outcome, Study Design
ROM: Range of Motion
MeSH: Medical subjects headings
RoB: Risk of bias
GRADE: Grading of Recommendations, Assessment, Development, and Evaluation
RCT: Randomized controlled trial
IG: Intervention group
CG: Control group
TUG: Timed up and Go test
BMI: Body Mass Index
6MWT: 6 Minutes-Walk Test
WOMAC: Western Ontario and McMasters Osteoarthritis Index
SF12: Short-form health survey 12
SF36: Short-form health survey 36
OKS: Oxford Knee Score
